# Moral Distress, Burnout, Turnover Intention, and Coping Strategies among Korean Nurses during the Late Stage of the COVID-19 Pandemic: A Mixed-Method Study

**DOI:** 10.1155/2024/5579322

**Published:** 2024-05-24

**Authors:** Jae Jun Lee, Hyunju Ji, Sanga Lee, Seung Eun Lee, Allison Squires

**Affiliations:** ^1^Department of Nursing, Graduate School, Yonsei University and Severance Hospital, Seoul 03722, Republic of Korea; ^2^Severance Hospital, Yonsei University Health System, Seoul 03722, Republic of Korea; ^3^Manning College of Nursing and Health Sciences, University of Massachusetts Boston, Boston, MA 02125, USA; ^4^Mo-Im KIM Nursing Research Institute, College of Nursing, Yonsei University, Seoul 03722, Republic of Korea; ^5^Rory Meyers College of Nursing, New York University, New York, NY 10010, USA

## Abstract

The COVID-19 pandemic has exacerbated the difficulties nurses face, resulting in higher turnover rates and workforce shortages. This study investigated the relationships between nurses' moral distress, burnout, and turnover intention during the last stage of the COVID-19 pandemic. It also explored the coping strategies nurses use to mitigate moral distress. Utilizing a mixed-method approach, this study analyzed data from 307 nurses caring for patients with COVID-19 in acute care hospitals through an online survey conducted in November 2022. Our data analysis encompassed quantitative methods, including descriptive statistics and path analysis, using a generalized structural equation model. For the qualitative aspect, we examined open-ended responses from 246 nurses using inductive content analysis. The quantitative findings revealed that nurses' moral distress had a significant direct effect on turnover intention. In addition, burnout significantly mediated the relationship between moral distress and turnover intention. Qualitative analyses contextualized the relationships uncovered in the quantitative analyses. The qualitative analysis identified various positive and negative coping strategies. Positive strategies included a commitment to minimize COVID-19 transmission risks, adopting a holistic approach amidst the challenges posed by the pandemic, voicing concerns for patient safety, engaging in continuous learning, and prioritizing self-care. Conversely, negative strategies involved adopting avoidance behaviors stemming from feelings of powerlessness and adopting a passive approach to one's role. Notably, some participants shifted from positive to negative coping strategies because of institutional barriers and challenges. The findings underscore the importance for hospital administrators and nurse managers to acknowledge the impact of the pandemic-related challenges encountered by nurses and recognize the link among moral distress, burnout, and turnover intention. It highlights the essential role of organizational and managerial support in fostering effective coping strategies among nurses to address moral distress.

## 1. Introduction

The COVID-19 pandemic has significantly heightened the challenges faced by nurses, resulting in increased turnover rates and looming concerns about workforce shortages [1, 2]. The Organisation for Economic Co-operation and Development reported that a substantial number of nurses considered leaving their positions during and after the pandemic [3]. Furthermore, the International Council of Nurses predicted a global shortage of up to 130,000 nurses, largely due to the pandemic [4]. Understanding the factors driving nurses' turnover intention is crucial to curtail these shortages. Traditional factors influencing nurse turnover include unfavorable work environments, unsupportive organizational cultures, high work demands, and insufficient social support [5, 6]. However, the pandemic has introduced novel concerns, such as COVID-19 fear, safety inadequacies, direct patient care challenges, personal infection risks, and moral distress [7]. Furthermore, moral distress, driven by pandemic-specific ethical dilemmas, has played a central role in nurses' contemplation of leaving their positions or professions.

Moral distress is an emotional reaction that arises when individuals are unable to act ethically owing to external constrains [8]. The global COVID-19 pandemic presented nurses with myriad of challenges, intensifying their experience of moral distress [9, 10]. Amid the pandemic, nurses were required to shoulder dual responsibilities—attending to basic patient needs and delivering specialized nursing care, especially as isolated COVID-19 patients lacked dedicated caregivers [10, 11]. The scarcity of personal protective equipment (PPE) endangered the direct care of COVID-19 patients [9]. These collective challenges heightened nurses' moral distress during the pandemic [9, 10, 12], fuelling their intention to depart from their nursing roles [7]. Previous research has identified the experience of moral distress among nurses during the COVID-19 pandemic as a significant contributor to turnover intention among nurses in Romania [13] and the US [14].

While previous studies have emphasized the impact of moral distress on nurses' intentions to leave [13–16], the underlying mechanisms explaining its nature remain ambiguous. Consistent findings suggest a correlation between chronic moral distress and the development of burnout [17, 18]. Facing recurring ethically distressing situations can result in nurses experiencing burnout symptoms such as exhaustion, cynicism, and decreased self-efficacy [19]. Exhaustion often stems from overwhelming workloads and when combined with perceptions of unfairness and a lack of rewards, a cynical perspective can develop [20]. Cynicism, which refers to psychological withdrawal from work, directly increases the propensity for turnover intention [20]. Given that the pandemic exacerbated moral distress through the intensified workloads and situations that undermined patients' quality of care and healthcare professionals' safety [10, 12, 18], it is plausible that this could precipitate burnout [17, 18]. Thus, we postulated that burnout could be a mediating factor in the relationship between moral distress and nurses' turnover intention.

Given the importance of moral distress on nurses' turnover intention, several studies have explored coping techniques. Most recommendations have focused on addressing the symptoms of moral distress through activities such as meditation, ensuring adequate sleep, listening to music [21], personal reflection, and informal conversations with colleagues [10, 22, 23]. However, these self-care strategies often align more with alleviating post-traumatic stress disorder symptoms than moral distress [24] and limited evidence supports their effectiveness in the context of moral distress. Furthermore, emphasizing these coping techniques may inadvertently frame moral distress as a challenge for individuals, implying that personal resilience is the primary solution. Considering the unique pressures of the COVID-19 pandemic, nurses might have utilized alternative coping strategies beyond the standard self-care approaches highlighted in prior studies. However, the strategies that nurses could have embraced to counteract moral distress during the pandemic remain underexplored, underscoring a critical gap in understanding and clarifying the need for further investigation into this area.

This study had the following three primary objectives: (1) to examine the relationship between moral distress and nurses' turnover intention; (2) to investigate whether burnout mediated this relationship; and (3) to identify the coping strategies employed by nurses to mitigate moral distress in the Korean healthcare context.

## 2. Materials and Methods

### 2.1. Study Design

This study used a concurrent mixed-methods design. Using an online survey, quantitative and qualitative data were simultaneously collected. This study was conducted as a part of the Global Consortium of Nursing and Midwifery Study, a multinational research consortium comprising 75 countries. For this study, we focused exclusively on data collected from Korean nurses, as only Korean dataset included items on moral distress.

### 2.2. Participants

Nurses who provided direct care to COVID-19 inpatients in acute care hospitals were invited to participate. Nurses with managerial roles were excluded because they do not provide direct care to patients in Korean hospitals. A total of 310 nurses completed the online survey, but data from three participants were excluded because they responded to all survey items with the same option number. Therefore, data from 307 nurses were analyzed. The ideal sample size for path analysis was at least 20 participants per variable [25]. Given that our study utilizes eight variables in path analysis, a minimum of 160 participants is required to ensure adequate statistical power and reliability of the results. Therefore, the sample size was deemed sufficient for this study.

### 2.3. Data Collection

In November 2022, data were collected from two Korean hospitals that experienced a significant influx of COVID-19 patient admissions. We collaborated with the nursing department of each hospital to facilitate participant recruitment. The nursing departments assisted in sending invitation emails containing secure links to an online survey, specifically to nurses caring for COVID-19 patients. Given that in Korea, only specific hospitals were designated as COVID-19 hospitals with designated isolation beds to contain the spread of the virus [26], we employed a snowball sampling method to augment our sample size and ensure its adequacy.

### 2.4. Measurements

#### 2.4.1. Demographic Characteristics

Participants' demographic information, including age, gender, educational level, hospital tenure, and monthly income, was collected.

#### 2.4.2. Moral Distress

Moral distress was measured using the COVID-19 Moral Distress Scale (COVID-MDS) [27]. The scale comprises 12 items across the following three domains: team/system (six items), patient (three items), and COVID-19 (three items). These domains capture the various sources of moral distress that nurses may experience while caring for COVID-19 patients [27].

After receiving approval from the original tool developers, the instrument was translated into Korean, following the translation guidelines of the Agency for Healthcare Research and Quality [28]. First, two bilingual nurses translated the scale into Korean. Subsequently, a nursing professor and three nurses with more than five years of nursing experience reviewed the translated items for their accuracy and appropriateness. A panel of five experts independently rated the relevance and accuracy of the translated items. The standard four-point Content Validity Index (CVI) rating scale, from 1 (not relevant) to 4 (highly relevant), was used to evaluate the conceptual relevance of each item, and the accuracy of the translation was answered as either yes or no [29]. Modified kappa statistics, which adjusted for chance agreement for CVI, were used to assess the relevance and accuracy of the translation [30]. The item-level CVI (I-CVI) for relevance was 1.0 for all 12 items. The modified kappa and the translation I-CVI (TI-CVI) were 0.76–1.0 and 0.80–1.0, respectively, indicating that the COVID-MDS is relevant for measuring moral distress in Korean nurse population [30]. Based on the results of the cognitive interviews with six nurses, the instrument was further refined and finalized. Subsequently, a confirmatory factor analysis (CFA) was performed on the data from 307 participants, and the results supported the three-factor model of the COVID-MDS. The CFA fit indices yielded favorable results, with a *χ*^2^(51) of 62.96, root mean square error of approximation of 0.03, standardized root mean square residual of 0.06, comparative fit index of 0.99, and Tucker–Lewis index of 0.99.

In line with the instructions provided by the tool developers [27], each item on the COVID-MDS was scored by multiplying the frequency ranging from 0 (never) to 3 (often) and the intensity ranging from 0 (no distress) to 3 (severe distress). This calculation yielded item scores ranging from 0 to 9. The total COVID-MDS score was calculated as the mean value for all the items' scores, with a higher value indicating a higher moral distress. The Cronbach's alpha value was 0.90 in the original study of the instrument [27] and the value was 0.90 in the present study.

#### 2.4.3. Burnout

Burnout was assessed using a single-item measure adapted from the Mini-Z Instrument, developed by Linzer et al. [31]. This measure has been validated against the Maslach Burnout Inventory [32], indicating good validity [31]. Responses were scored on a 5-point scale ranging from 1 (no symptoms of burnout) to 5 (completely burned out). Consistent with previous studies [33], scores over 2 were considered indicative of burnout.

#### 2.4.4. Turnover Intention

Turnover intention was measured using a single-item question that asked nurses whether they were considering leaving their current job within the next year. The response options provided were no, maybe, or yes. In line with previous research [34], we recategorized the responses as a binary variable as follows: 0 (no turnover intention) and 1 (turnover intention, including those who answered either maybe or yes).

#### 2.4.5. Strategies to Mitigate Moral Distress

Participants were asked to answer an open-ended question regarding the strategies they employed to alleviate moral distress while caring for COVID-19 patients. The question was presented as a free-text response, allowing the participants to provide as much detail as they wished. Since online surveys that incorporated open-ended questions were shown to be a suitable method for collecting qualitative data during the COVID-19 pandemic given their flexibility and accessibility [11], we utilized this method.

### 2.5. Ethical Considerations

This study received ethical approval from the Institutional Review Board of Yonsei University Health System (#4-2022-0675). The online survey introduction page provided participants with information about the purpose of the study, eligibility criteria, study duration, and the estimated time required to complete the survey. Participation in the study was voluntary, and participants had the freedom to withdraw at any point without any consequences. Written informed consent was adapted to the online format of our survey. At the beginning of the survey, participants encountered mandatory consent checkboxes. By selecting these checkboxes, participants indicated their informed consent to participate in this study. As a token of appreciation for their participation, all participants were offered a gift card worth approximately US$12.

### 2.6. Data Analysis

For the quantitative data, descriptive statistics were employed to examine the sample characteristics and key study variables. Path analysis was conducted using generalized structural equation modeling (GSEM), controlling for covariates, to investigate the mediating effect of burnout on the relationship between moral distress and turnover intention. Given that GSEM is suitable for path analyses involving binary variables [35], we chose this analytical method because burnout and turnover intention are binary variables. Bootstrapping based on 5,000 replications was performed to test the significance of direct and indirect effects [36]. All statistical analyses were performed using STATA 16.0 with the statistical significance level set at 0.05.

For the qualitative data analysis, we adopted the inductive content analysis method proposed by Graneheim and Lundman [37]. Of the 307 survey participants, 246 responded to the open-ended questions, yielding a dataset of free-text data from 246 participants. The analysis was conducted by three trained coders, who engaged in iterative discussions to address any discrepancies and ensure consistency in the coding process. Prior to commencing the analysis, all coders thoroughly familiarized themselves with the participants' responses by carefully reading through the data. In total, 344 meaning units were identified from the responses. Subsequently, the coders condensed the meaning units and independently assigned codes to them. Through ongoing discussions and comparison of coding outcomes, any discrepancies in coding were resolved through consensus among the coders. Similar codes were grouped into subcategories, which were then merged to form major categories. This meticulous process was employed to uphold the rigor and quality of the data analysis, ensuring reliability and validity of the findings.

## 3. Results

### 3.1. Description of the Participants

A total of 307 nurses who had cared for COVID-19 patients participated in this study. The majority of participants (*n* = 289, 94.1%) were female, with an average age of 32.1 (SD = 6.5) years. Most participants held a bachelor's degree (*n* = 245, 79.8%) and their mean length of hospital tenure was 89.4 (SD = 66.2) months. More than half of the participants (*n* = 174, 56.7%) reported experiencing burnout, and 41.4% (*n* = 127) reported an intention to leave their current jobs (Table 1).

### 3.2. Moral Distress

Table 2 illustrates the scores for the moral distress subdomains and the summary scores. The summary score of moral distress was 3.0 (SD = 1.8), with the COVID-19 domain (mean = 3.3, SD = 2.3) exhibiting the highest score among the three domains. Nurses reported experiencing the highest levels of moral distress when assigned an unsafe number of patients. This was followed by caring for COVID-19 patients who presented with a transmission risk and caring for patients who had to be hospitalized without their family members.

### 3.3. The Relationships between Moral Distress, Burnout, and Turnover Intention

**Figure 1:**
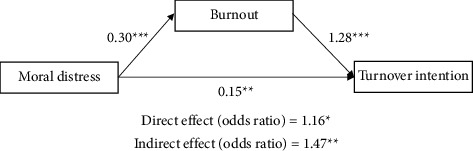
Results of path analysis using generalized structural equation modelling. Age, gender, educational level, hospital tenure, and monthly income were adjusted for in the model, and results were bootstrapped with 5,000 repetitions. The unstandardized coefficients were used to express the correlation between variables. The direct and indirect effects are presented as odds ratios at the bottom of the figure. ^*∗*^*p* < 0.05, ^*∗∗*^*p* < 0.01, ^*∗∗∗*^*p* < 0.001.

Figure 1 illustrates the associations between moral distress and turnover intention through burnout, while accounting for participants' age, gender, educational level, hospital tenure, and monthly income. Moral distress was positively associated with burnout (coefficient = 0.30, *p* < 0.001) and turnover intention (coefficient = 0.15, *p* < 0.01). Burnout was significantly related to turnover intention (coefficient = 1.28, *p* < 0.001). In addition, burnout significantly mediated the relationship between moral distress and turnover intention (OR = 1.47, 95% bias-corrected CI = 1.13–1.92).

### 3.4. Coping Strategies to Mitigate Moral Distress

The qualitative data analysis yielded three main categories and eight subcategories regarding nurses' moral distress coping strategies (Table 3). The three main categories were positive, negative, and shifting from positive to negative coping strategies. The first category, positive-coping strategies, encompasses five subcategories that highlighted the proactive strategies employed by nurses to address moral distress and foster personal growth. The second category, negative-coping strategies, consisted of two subcategories that clarified the passive and negative ways in which nurses coped with moral distress. The third category, shifting from positive to negative coping strategies, describes how some nurses initially utilized positive coping strategies, but subsequently shifted towards negative ones.

#### 3.4.1. Positive-Coping Strategies

*(1) Being Committed to Minimizing COVID-19 Transmission Risks*. Nurses adhered to stringent protocols and measures to alleviate moral distress stemming from the potential transmission risks inherent in caring for COVID-19 patients. They strictly adhered to institutional policies by properly wearing PPE and actively adhering to the quarantine guidelines. The participants strictly prohibited contact between patients to prevent transmission and took precautions to avoid personal contamination.

*“I strictly followed the quarantine guidelines to ensure both our patients and we remained shielded from infection.” (P57)*

*“I've been trying my best to prevent virus transmission and avoid contamination.” (P179)*

*(2) Adopting a Holistic Approach Amidst COVID-19 Challenges*. Several nurses stated that they tried to provide more comprehensive care for their patients by meeting patients' needs, focusing on patient education, and referring to patients' relevant resources to mitigate their moral distress. Participants noted that they attempted to provide nursing care that addressed the unique needs of each patient. Some participants stated that they carefully assessed each patient's symptoms and focused on the fundamental nursing care that they might not usually provide, such as oral hygiene and eye care. Notably, COVID-19 patients experienced isolation without the presence of family caregivers, and nurses took on the responsibility of frequently monitoring and assisting patients with their personal hygiene and safety. Many respondents also stated that they educated or counseled patients and their family members frequently to help enhance their understanding of their health status and the treatment journey. One nurse shared her practice of providing daily briefings to patients to ensure that they were informed about their treatment progress. Another nurse shared that when patients decided whether to continue life-sustaining treatment, she explained the details of each treatment to help them make informed decisions. In addition, some nurses actively directed patients to resources, connecting those with financial constraints to hospital aid programs or even seeking extra assistance for their patients who could not afford necessary treatments.

*“I completed other tasks as quickly as possible and devoted time to basic nursing care for patients, including position change, oral care, eye care, and lung care.” (P279)*

*“I made an effort to explain both the current physical condition and the purposes of treatments to patients. I aimed to briefly summarize the patients” progress of each day and share any improvements with them and their caregivers.” (P124)*

*“When a patient refused treatment due to financial constraints, I sought ways to assist the patient.” (P59)*

*(3) Voicing Concerns for Patient Safety*. Nurses noted that they advocated for patient safety by voicing their concerns to supervisors or physicians to alleviate their moral distress. They proactively approached their supervisors, urging an increase in the number of nurses assigned to units to enhance the quality of nursing cars. These nurses recognized the detrimental effects of nursing shortages on patient outcomes and emphasized the importance of adequate staffing to maintain high-quality care. In addition, nurses took it upon themselves to clarify and question physicians' orders that appeared inappropriate or lacked sufficient evidence supporting their effectiveness. They recommended alternative approaches and advocated for better decision-making to ensure their patients' best interests. One nurse saw a patient suffer immensely from an unnecessary and improper prescription and spoke to both a resident and attending physician, urging a change in prescription practices.

*“I asked my manager to increase the number of nurses working in our department to ensure our patients received safer care.” (P167)*

*“The most harrowing experience for me was witnessing a cancer patient, already terminally ill, die without dignity. Despite his decision to refuse life-sustaining treatment, the patient had to suffer because his physician prescribed numerous tests for him and did not prescribed the appropriate pain meds. I have become more vocal, ensuring my opinions and concerns are heard, even if it means addressing an attending physician directly.” (P149)*

*(4) Continuous Learning*. Participants strongly emphasized the importance of continuous learning to adapt to the challenges posed by the emergence of a new disease and to provide evidence-based care to their patients. They sought educational seminars and training opportunities, recognizing the importance of staying updated and informed in their practice. Some participants elected to go further with their continuous learning by pursuing graduate education to deepen their clinical knowledge and professional development.

*“I always study to find the latest evidence for my practice.” (P305)*

*“Due to frequent reassignments to units where I was tasked with unfamiliar work, I felt the need for comprehensive knowledge, prompting my decision to attend a graduate nursing program.” (P84)*

*(5) Engaging in Self-Care*. The participants sought strategies to relax and maintain their emotional wellbeing. Prayer, meditation, and deep breathing were commonly identified for managing stress. Some nurses described seeking professional help from psychiatrists and psychologists to cope with the stress and emotional demands of their work. Nurses reported that support from family and friends also helped reduce moral distress.

*“I used relaxation therapy, such as deep breathing and meditation.” (P76)*

*“I talked to a psychiatrist to reduce my moral distress.” (P158)*

#### 3.4.2. Negative-Coping Strategies

*(1) Adopting Avoidance Behaviors due to Feelings of Powerlessness*. Some nurses expressed feelings of powerlessness when confronted with morally distressing situations, leading them use avoidance as a coping mechanism. They believed that, as nurses, they lacked the authority to effect meaningful changes and felt that their opinions would be disregarded by hospital managers. Consequently, the nurses would rationalize their inaction in morally distressing situations and attempt to distance themselves from such experiences by striving to forget or suppress them.

*“In practice, there is nothing a nurse can do to solve the problems. Even when we voice our concerns, they tend to be overlooked, making us feel that our opinions hold no weight.” (P197)*

*“I believed that, as a nurse, I lacked the authority to bring about real change, so I tried to avoid those distressing moments.” (P106)*

*(2) Becoming Passive in One's Role*. Some participants adopted a passive approach to work in response to stressful situations. They described feeling discouraged from taking proactive measures because they believed that the hospital did not value or accept their proactive attitude. Consequently, these participants limited themselves to completing only the assigned tasks without engaging in critical thinking or going beyond their assigned responsibilities. In addition, they shared instances in which they ignored minor patient demands, considering them too burdensome or requiring excessive effort. Consequently, these participants adopted a formal, business-like approach when interacting with patients and focused solely on completing the basic and necessary tasks.

*“I began to just stick to my assigned tasks without taking on any unnecessary duties.” (P207)*

*“I only spoke when it was absolutely necessary.” (P260)*

*“Given the overwhelming workload, I felt I could not change my situation, so I often overlooked patients” minor requests.” (P241)*

#### 3.4.3. Shifting from Positive- to Negative-Coping Strategies

Some participants reported a shift in their approach to managing their moral distress. Initially, they attempted to employ positive strategies such as referring patients to external support services and reporting inappropriate situations to their supervisors; however, their ability to effectively address moral distress was hindered after encountering challenges and obstacles within their institutions. The experience of encountering obstacles and a lack of organizational support profoundly impacted the nurses, leading to a sense of resignation, which resulted in their decision to leave their current job in some cases.

*“Initially, I believed it was part of my role to seek external resources for patient recovery. But without any support, compensation, encouragement, or rewards, and even facing criticism for taking an initiative, I eventually stopped putting in the extra effort.” (P16)*

*“I used to communicate my concerns to my manager or report them to the relevant department, but in the end, I decided to leave this job.” (P96)*

## 4. Discussion

To the best of our knowledge, this is the first study to examine the relationship among moral distress, burnout, and turnover intention among nurses and investigate the coping strategies that were employed to mitigate this distress within the Korean healthcare context using a mixed-methods approach. Our quantitative analyses revealed a significant association between higher levels of moral distress and increased turnover intention among nurses. Also, burnout was identified as a mediator in this relationship. Moreover, our qualitative analysis identified the various coping strategies nurses used to manage moral distress.

During the COVID-19 pandemic, nurses faced challenging situations leading to significant moral distress, as underscored by our findings and corroborated in a US study [38]. Both studies identified the increased patient care volume and potential infection risk to the nurses' families as the primary contributors to this distress. The global PPE shortage could have intensified this distress, leaving many nurses unprotected, consequently heightening their anxiety [9, 10, 21, 38]. When planning for future pandemic response implementation, healthcare organizations must ensure adequate PPE supplies, enforce stringent safety measures [10, 38], and provide clearly communicated policies and guidelines [38] to minimize the risk of nurses experiencing avoidable moral distress.

Our findings build on previous research [13–16] and demonstrated a direct association between nurses' moral distress and turnover intention. Nursing professionals often derive motivation from the intrinsic value of their roles, commitment, and passion for patients [39]. However, when they encounter situations that conflict with their ethical practices, their professional satisfaction diminishes, leading to increased turnover intentions [40]. This issue became particularly apparent during the COVID-19 pandemic when nurses confronted moral distress due to barriers hindering high-quality care [10]. A prior study demonstrated that during the pandemic, nurses experienced higher levels of stress, leading to an increased intention to leave their positions, compared to prepandemic times [41]. In addition, consistent with previous findings [17, 18, 42], we also found that burnout significantly mediated the relationship between moral distress and turnover intention, which aligns with the moral distress model [43], positing that unresolved moral distress heightens burnout, further increasing their inclination to leave the nursing profession. Thus, effectively addressing moral distress can be a strategy for reducing burnout and nurse turnover [13].

Our qualitative analysis revealed a range of coping strategies that nurses adopted in situations causing them moral distress, ranging from positive to potentially negative approaches. In line with previous research [10, 21–23], we found that nurses engaged in self-care practices, such as prayer and meditation, to manage their distress. Positive-coping strategies in our study were marked by a proactive commitment to patient care. This commitment is evident in their strict adherence to infection prevention guidelines, reflecting their dedication to the safety and their patients' welfare. Moreover, nurses frequently exceeded their standard duties to ensure that patients experienced personalized care in the absence of family caregivers as they recognized the isolating circumstances of COVID-19 hospitalizations. They also actively voiced concerns about enhancing the quality of patient care. This deep commitment might be attributed to the unique emotional challenges posed by the COVID-19 pandemic, as suggested by Ahokas and Hemberg [44].

However, recognizing the negative-coping strategies driven by feelings of powerlessness and a lack of organizational and managerial support is equally important. Our findings revealed that nurses shifted from positive- to negative-coping strategies when they felt inadequate organizational support, underscoring the importance of organizational and managerial support. These insights emphasize the urgency for hospital administrators and nurse managers to genuinely value frontline nurses' feedback. Previous research has emphasized the significance of administrative support [45], suggesting that nurse managers should establish clear policies and guidelines to promptly address nurses' concerns [38]. In addition, addressing the issue of moral distress during meetings can demonstrate to nursing staff that their wellbeing is a priory for their managers [44]. Moreover, nurse managers' inclusive leadership can be instrumental in supporting nurses experiencing moral distress because such leaders foster open communication, value diverse perspectives, and prioritize shared decision-making [46].

The findings also help conceptually delineate avoidable and unavoidable moral distress when implementing a pandemic response. Avoidable moral distress is associated with the structural and organizational factors related to implementing a pandemic response. These would include, but are not limited to, resource management, communication patterns, nurse staffing and managerial leadership and how organizational culture sets the tone for pandemic response implementation [47]. We theorize that avoidable moral distress is more likely to contribute to burnout because structural and organizational factors are perceived as being controllable and addressable than those associated with unknowns.

Unavoidable moral distress is that associated with the unknowns and uncertainties of working during a pandemic, such as the nature of the disease, its effects on patients and their caregivers, evolving treatments, and other factors [10]. Unavoidable moral distress is where resilience and coping strategies may play a stronger role in mitigating the stressors of working during a pandemic, along with perceived mental health and social support of nurses working on the frontlines of pandemics [10, 48, 49]. Conceptually distinguishing between the factors driving moral distress as experienced by nurses working the frontlines of pandemics is important for optimizing interventions and support services for them. It may further help develop our understanding of the drivers of turnover as well. Thus, there is a need for further research in this area.

This study had several limitations. First, we used a cross-sectional design; therefore, the causal relationships among the study variables cannot be determined. Second, the data were collected using self-reported questionnaires; thus, there might have been a potential desirability bias because some participants could have hesitated to provide honest answers to certain questions [50]. Third, the study only included nurses working in Korean acute care hospitals, which may not be generalized to other healthcare settings. Finally, although the open-ended questions allowed us to identify the coping strategies used by nurses, contextual information regarding the pre- and postapplication of these strategies was not collected. Therefore, future qualitative studies using alternative methods, such as in-depth interviews, could provide detailed and insightful responses from the participants.

## 5. Conclusions

During the COVID-19 pandemic, the immense challenges faced by healthcare professionals, particularly nurses, were undeniable. This study investigated the challenges faced by nurses in Korea, highlighting the impact of moral distress on turnover intention, with burnout as a significant mediator. The unique aspects of pandemic care exacerbated moral distress, prompting nurses to adopt various coping strategies. Although some of these strategies were constructive, a shift from positive to negative mechanisms was evident in the absence of organizational and managerial support, highlighting the importance of robust institutional support. Our findings could provide valuable insights into leadership practices during future crises.

## Figures and Tables

**Table 1:** General characteristics of the study participants (*N* = 307).

Variables	Mean (SD)	*n* (%)
Age (years)	32.1 (6.5)	
Gender		
Male		18 (5.9)
Female		289 (94.1)
Educational level		
Associate's degree		38 (12.4)
Bachelor's degree		245 (79.8)
Master's degree		24 (7.8)
Hospital tenure (months)	89.4 (66.2)	
Monthly income (10,000 Korean won)	379.9 (94.4)	
Burnout		
Yes		174 (56.7)
No		133 (43.3)
Turnover intention		
Yes		127 (41.4)
No		180 (58.6)

*Note*. SD: standard deviation.

**Table 2:** COVID-19-related moral distress.

Domain	Frequency	Intensity	Multiplied score
	Mean (SD)	Mean (SD)	Mean (SD)
Patient domain	1.4 (0.9)	1.4 (1.0)	2.4 (2.0)

Being asked to provide and continue aggressive and potentially futile treatments when I believe it is not in the best interest of the patient	1.6 (0.8)	1.5 (1.0)	2.7 (2.5)
Witnessing orders for unnecessary or inappropriate care that do not adequately address patient needs	1.2 (0.8)	1.2 (1.0)	2.0 (2.3)
Providing care to patients who have not been adequately informed or included in decisions regarding their own care	1.5 (0.9)	1.4 (1.0)	2.6 (2.4)

Team/system domain	1.7 (0.9)	1.5 (1.0)	3.1 (2.0)

Experiencing poor communication between members of the care team that adversely affects patient care	1.7 (0.7)	1.6 (0.9)	3.0 (2.4)
Being assigned an unsafe number of patients to care for at once considering the acuity level for each patient assigned to me	2.1 (0.8)	1.9 (0.9)	4.4 (3.0)
Attempting to deliver a high standard of care with limited time, supplies, and resources	1.7 (0.9)	1.5 (1.0)	3.1 (2.8)
Using technology and documentation that burdens me and compromises patient care	1.6 (0.8)	1.5 (0.9)	2.8 (2.5)
Witnessing or experiencing uncivil behavior among members of the care team	1.8 (0.9)	1.6 (1.0)	3.2 (2.9)
Witnessing a lack of respect among the healthcare team for patients from vulnerable populations or minority groups	1.1 (0.9)	1.1 (1.1)	1.8 (2.3)

COVID-19 domain	1.8 (0.9)	1.5 (1.0)	3.3 (2.3)

Caring for patients who must experience hospitalization without family presence	2.1 (0.8)	1.6 (0.9)	3.7 (2.7)
Caring for patients who die during a hospitalization without family and/or clergy present	1.3 (1.0)	1.3 (1.1)	2.4 (2.7)
Caring for COVID‐19 patients that presents a transmission risk to you or your family/household	2.0 (0.9)	1.7 (1.0)	3.8 (2.9)

Summary score	1.6 (0.9)	1.5 (1.0)	3.0 (1.8)

*Note*. SD: standard deviation.

**Table 3:** Coping strategies to mitigate moral distress.

Categories	Subcategories
Positive-coping strategies	Being committed to minimizing COVID-19 transmission risks
	Adopting a holistic approach amidst COVID-19 challenges
	Voicing concerns for patient safety
	Continuous learning
	Engaging in self-care

Negative-coping strategies	Adopting avoidance behaviors due to feelings of powerlessness
	Becoming passive in one's role

Shifting from positive- to negative-coping strategies	—

## Data Availability

The data used to support the findings of this study are not available because the participants did not provide written consent for their data to be shared publicly.
